# 3D Printing of Polymer-Bonded Rare-Earth Magnets With a Variable Magnetic Compound Fraction for a Predefined Stray Field

**DOI:** 10.1038/s41598-017-09864-0

**Published:** 2017-08-25

**Authors:** Christian Huber, Claas Abert, Florian Bruckner, Martin Groenefeld, Stephan Schuschnigg, Iulian Teliban, Christoph Vogler, Gregor Wautischer, Roman Windl, Dieter Suess

**Affiliations:** 1 Physics of Functional Materials, University of Vienna, 1090 Vienna, Austria; 2 Christian Doppler Laboratory for Advanced Magnetic Sensing and Materials, 1090 Vienna, Austria; 3 Magnetfabrik Bonn GmbH, 53119 Bonn, Germany; 4 Department of Polymer Engineering and Science, Montanuniversitaet Leoben, 8700 Leoben, Austria

## Abstract

Additive manufacturing of polymer-bonded magnets is a recently developed technique, for single-unit production, and for structures that have been impossible to manufacture previously. Also, new possibilities to create a specific stray field around the magnet are triggered. The current work presents a method to 3D print polymer-bonded magnets with a variable magnetic compound fraction distribution. This means the saturation magnetization can be adjusted during the printing process to obtain a required external field of the manufactured magnets. A low-cost, end-user 3D printer with a mixing extruder is used to mix permanent magnetic filaments with pure polyamide (PA12) filaments. The magnetic filaments are compounded, extruded, and characterized for the printing process. To deduce the quality of the manufactured magnets with a variable magnetic compound fraction, an inverse stray field framework is developed. The effectiveness of the printing process and the simulation method is shown. It can also be used to manufacture magnets that produce a predefined stray field in a given region. This opens new possibilities for magnetic sensor applications. This setup and simulation framework allows the design and manufacturing of polymer-bonded permanent magnets, which are impossible to create with conventional methods.

## Introduction

Additive manufacturing is an affordable, rapid technique to manufacture models, tools, prototypes, or end products. The production is carried out directly from formless (liquids, powders, etc.) or form-neutral (tape, wire) material, mostly by means of thermal or chemical processes. No specific tools are required for a specific object with a possibly complex shape. A well-established additive manufacturing method is the fused deposition modeling (FDM) technology. FDM, also referred to as 3D printing, is a process that uses wire-shaped thermoplastic filaments. The filament is heated just above its softening point with the aid of a moving heated extruder. Molten thermoplastic is pressed out of the printer head nozzle and builds up the object layer by layer on the already solidified material on the building platform^1^. Since 3D printers are nowadays affordable for end-users, a boom of new possibilities have been triggered^2^. 3D print technology is a fast-growing field for single-unit production, and it allows to produce structures that have been difficult or impossible to build before.

NdFeB magnets are mainly divided into sintered and polymer-bonded magnets. On the one hand, sintered magnets have the highest maximum energy product (*BH*)_max_; on the other hand, polymer-bonded magnets enable the manufacturing of complex shapes and magnetization structures, but with a lower (*BH*)_max_^3^. Therefore, they are widely used wherever product cost is a major consideration over magnetic performance^4^. Bonded magnets offer a wide application range, from sensor to actuator applications^5^.

Polymer-bonded magnets are composites with permanent-magnet powder embedded in a polymer binder matrix. Hard magnetic particles, ferrite (e.g., Sr, Ba), and rare-earth materials (e.g., NdFeB) with a volume filler content between 40–65 vol.% are inserted. These compounds can be further processed with injection molding or extrusion^6^. The NdFeB particles for the compounds are produced by a melt spinning process. To achieve better rheological behavior, spherical particles are preferred, which can be produced by an inert gas atomization process. To reduce assembling costs and reach more flexibility, magnetically isotropic powder is preferred. The high filler content increases the viscosity of the melted compound^7^. To avoid clogging of the nozzle, the matrix polymer should be of a high flowable material; good mechanical properties are an important aspect, too. Polyamides (PA6, PA11, and PA12) have a good combination of these qualities.

Recently, it was shown that an end-user 3D printer can be used to print polymer-bonded rare-earth magnets with a complex shape^8^. A prefabricated magnetic compound (Neofer^®^ 25/60p) from Magnetfabrik Bonn GmbH has been used. It consists of 90 wt.% NdFeB grains in a PA11 matrix. The effectiveness of this printing method is demonstrated by fabrication of a magnet with a complex shape that is known to produce a specific stray field above the printed magnet. Structures with a size of under 0.8 mm, and a layer height of under 0.1 mm are possible. Contrary to the well-established, affordable, accurate, and high-resolution end-user 3D printing technology, big area additive manufacturing (BAAM) of large-scale NdFeB magnets is presented in ref. 9. The BAAM method operates within the same principle as a conventional 3D printer. An advantage of this method is the possibility to manufacture large-scale objects, disadvantages are the high system costs and printing of fine structures is impossible due to the large printer nozzle size.

However, at the moment, no other single-unit manufacturing technologies are available for the production of magnets with complex shapes; nor is the opportunity to fabricate objects without material waste, and a minimum amount of source material. This can be an important aspect for the reduction of rare-earth elements in permanent magnets^10^.

In this work, a method to manufacture polymer-bonded permanent magnets with a variable magnetic compound fraction ${\varrho }_{m}$ along the printing direction is presented, where ${\varrho }_{m}$ is defined as the fraction of the magnetic compound material from the entire volume that is pressed out of the heated printer nozzle. The filler fraction of the magnetic material is proportional to the remanence *B*_*r*_. This can be used to shape the magnetic field without changing the topology of the object. First, the effectiveness of the method is shown. Furthermore, an inverse stray field method based on finite elements has been developed, which allows to deduce the magnetic compound fraction and magnetization distribution of the magnet from stray field measurements. This method can be used to evaluate the quality of the printed magnets. Moreover, the inverse method allows us to find an optimal magnetization density distribution for a given target field.

## Methods

### Printing/Simulation Models

The models for the 3D printing process and the simulations are created in Salome 7.6. Meshing of the simulation models is performed in Salome 7.6 with the Netgen algorithm and tetrahedron elements (Supplementary Fig. 1)^11^. Converting the.med Salome output file to the FEniCS.xml format is performed with Gmsh 2.10.1. The STL file for the 3D printing process is sliced with Slic3r 1.3.0. The resulting G-code was further modified by a customized Python script to print objects with a layer-dependent magnetic compound fraction distribution.

### Stray Field Simulation

In a simply connected domain without current, the stray field ${\overrightarrow{h}}_{{\rm{m}}}$ of a magnetic body is given by^12^

1 $${\overrightarrow{h}}_{{\rm{m}}}=-\nabla u$$

with the magnetic scalar potential *u* being subject to the Poisson equation

2 $${\rm{\Delta }}u=\nabla \cdot \overrightarrow{M}$$

and open boundary conditions

3 $$u(\overrightarrow{x})={\mathscr{O}}\mathrm{(1/|}\overrightarrow{x}|)\quad {\rm{for}}\quad \overrightarrow{x}\to \infty \mathrm{.}$$

where $\overrightarrow{M}$ is the magnetization at a position $\overrightarrow{x}$. We apply the finite-element method based on FEniCS2016.1 for the solution of the Poisson equation^13^. The open boundary conditions are approximated with a simple truncation approach, i.e. the nonmagnetic external region is also meshed to a certain extend and the potential is set to zero at the outer boundary by means of Dirichlet boundary conditions. In order to get reasonable results, we choose the external region to be 5 times larger then magnetic region in each spatial dimension as suggested in ref. 14.

We use piecewise affine and globally continuous functions for the discretization and solve (2) on complete region ${\rm{\Omega }}={{\rm{\Omega }}}_{m}\cup {{\rm{\Omega }}}_{h}\cup {{\rm{\Omega }}}_{a}$ with Ω_*m*_, Ω_*h*_, and Ω_*a*_ being the magnetic region, the measurement region, and the air region respectively.

### Printer

For the printing process, the conventional end-user 3D printer Builder from Code P is chosen (Supplementary Fig. 2). This printer works by use of the fusing deposition modeling (FDM) principle. This system creates the object layer by layer by a meltable thermoplastic. It has a maximum building size of 220 × 210 × 164 mm^3^ (L × W × H). Structures with a layer height resolution between 0.05 and 0.3 mm can be printed. The printing speed ranges from 10 to 80 mm/s, and the traveling speed ranges from 10 to 200 mm/s. The nozzle diameter is 0.4 mm, and by the means of a dual feed extruder, two different compound materials can be mixed, or a defined region of the object can be printed with different materials. The maximum nozzle temperature is 260 °C. For a better adhesion of the printed objects, the printer bed can be heated up to 80 °C. The optimal printing parameter for our setup and our magnetic compound filaments are listed in Table 1.

**Table 1:** Best empirically found printer parameters for the magnetic compound material.

Parameter	Value
Extruder temp.	260 °C
Layer height	0.15 mm
Printer speed	20 mm/s
Fill density	100%
Fill pattern	Rectilinear
Bed adhesion	Kapton tape with a layer of Polyvinyl acetate (PVA)
Bed temp.	60 °C

### Filament Manufacturing

The polymer-bonded magnetic compound consists of polyamide 12 (PA12; also called as Nylon 12) from Polyking (221-TR) and magnetically isotropic powder MQP-S-11-9 with the chemical composition NdPrFeCoTiZrB from Magnequench Corporation. These source materials are compounded and extruded into suitable filaments in the desired ratio of 85 wt.% MQP-S-11-9 powder and 15 wt.% PA12. The extrusion is performed at University of Leoben with a Leistritz ZSE 18 HPe-48D twin-screw extruder. The materials are dried at 80 °C for 8 hours. The four heating zones of the twin-screw are temperate. The feed section is the coolest with 80 °C, and the temperature increases up to the shaping die, which has a temperature of 260 °C. The round orifice of the die has a diameter of 1.75 mm. The hot extrudate is hauled off and cooled by a cooled conveyor belt. The diameter and tolerances of the filament are controlled by a Sikora Laser Series 2000 diameter-measuring system. The extrusion speed is adjusted to get a filament with a diameter of 1.75 mm. The manufactured filament is spooled with a diameter of around 0.5 m to avoid breaking of the brittle magnetic filaments.

### Material Characterization

The fraction of NdFeB particles in the PA12 matrix is measured by thermogravimetric analysis (TGA). The model TGA 2050 from TA-Instruments has a resolution of 0.2 μg and a temperature range of 25–1000 °C. In our case, a heating rate of 10 K/min and a nitrogen atmosphere to avoid oxidation of the particles are used. TGA measurement yields a filler content of 85 wt.%. Hysteresis measurements are performed for different magnetic compound fractions ${\varrho }_{m}$. With the dual extruder of our printer, cubes with a size of 7 mm are printed and afterwards post processed to obtain cubes with a length of *a* of 5 ± 0.02 mm. To minimize the statical error, 3 cubes are printed with the same printing parameter. The hysteresis is measured by Pulsed Field Magnetometry (PFM) (Hirst PFM11)^15, 16^. All measurements are carried out 3 times with the same parameters - temperature of 297 K and a magnetic field up to 4 T peak field. The internal field is ${H}_{{\rm{int}}}={H}_{{\rm{ext}}}-N/{\mu }_{0}J$, where *H*_ext_ is the external field, *N* is the average demagnetisation factor for a cube (*N* = 1/3)^17^, and *J* is the material polarization. The morphology of the NdFeB particles is identified by Scanning Electron Microscope (FEI Quanta 200 FEG) images. The samples are Au-coated with a Sputter Coater Quorum (Q150T S). The particles are of spherical shape with a diameter of approximately 45 ± 20 μm (Supplementary Fig. 3).

### Magnetization

The objects with a variable magnetic compound fraction are magnetized inside an electromagnet. It is a self-built water-cooled electromagnet, and it is powered by a low-voltage power supply (Siemens NTN 35000-200). Maximum output current is 150 A with an operating voltage of 200 V. This setup has a maximum magnetic flux density inside the electromagnet of 1.9 T in permanent operation mode. The gap between the pole shoes is 50 mm.

### Stray Field Measurement

To measure the stray field of the printed permanent magnet, the 3D printer is upgraded to a full 3D magnetic flux density measurement system. As a sensing device, a 3D Hall sensor TLV493D-A186 from Infineon is used. A Genuino 101 microcontroller is programmed to read out the components of $\overrightarrow{B}$ with a frequency of 3 kHz. The sensor has a measurement range of ±130 mT, and a measured detectivity of 40 μ﻿T/$\sqrt{{\rm{Hz}}}$ for static magnetic fields. A Python script controls the movement of the 3D printer and saves the stray field measurement data for the actual position of the sensor. This setup has a spatial resolution of 0.05 mm along the *z*-axis and 0.1 mm along the *x*- and *y*-axis. All stray field measurements are performed at least 3 times, and the average was formed. To skip an elaborate adjustment and alignment of the sensor, a calibration method on a detailed stray field simulation is used^8^. With this method, the angles, sensitivity, and the offset of the sensor can be calibrated. In our case, the sensor is simply attached to the extruder head with a self-printed suspension without any adjustment (Supplementary Fig. 4). With this setup, the stray field can be scanned in 1D, 2D, and 3D around a complex magnetic structure.

## Results

### Predefined Magnetic Compound Fraction

A mixing extruder of an end-user 3D printer has the possibility to mix two or more materials during the printing process. In this article, the mixing extruder is used to mix magnetic compound material with pure commercial PA12. The magnetic compound consists of 85 wt.% NdFeB particles inside a PA12 matrix. Commercial magnetically isotropic powder MQP-S-11-9 with the chemical composition NdPrFeCoTiZrB from Magnequench Corporation is used. The powder is produced by employing an atomization process, followed by heat treatment. The particles are of spherical morphology with a diameter of approximately 45 ± 20 μm (Supplementary Fig. 1). The magnetic compound is extruded into suitable filaments with a diameter of 1.75 ± 0.1 mm and a magnetic filler content of 85 wt.% and 43 vol.%, respectively.

**Figure 1:**
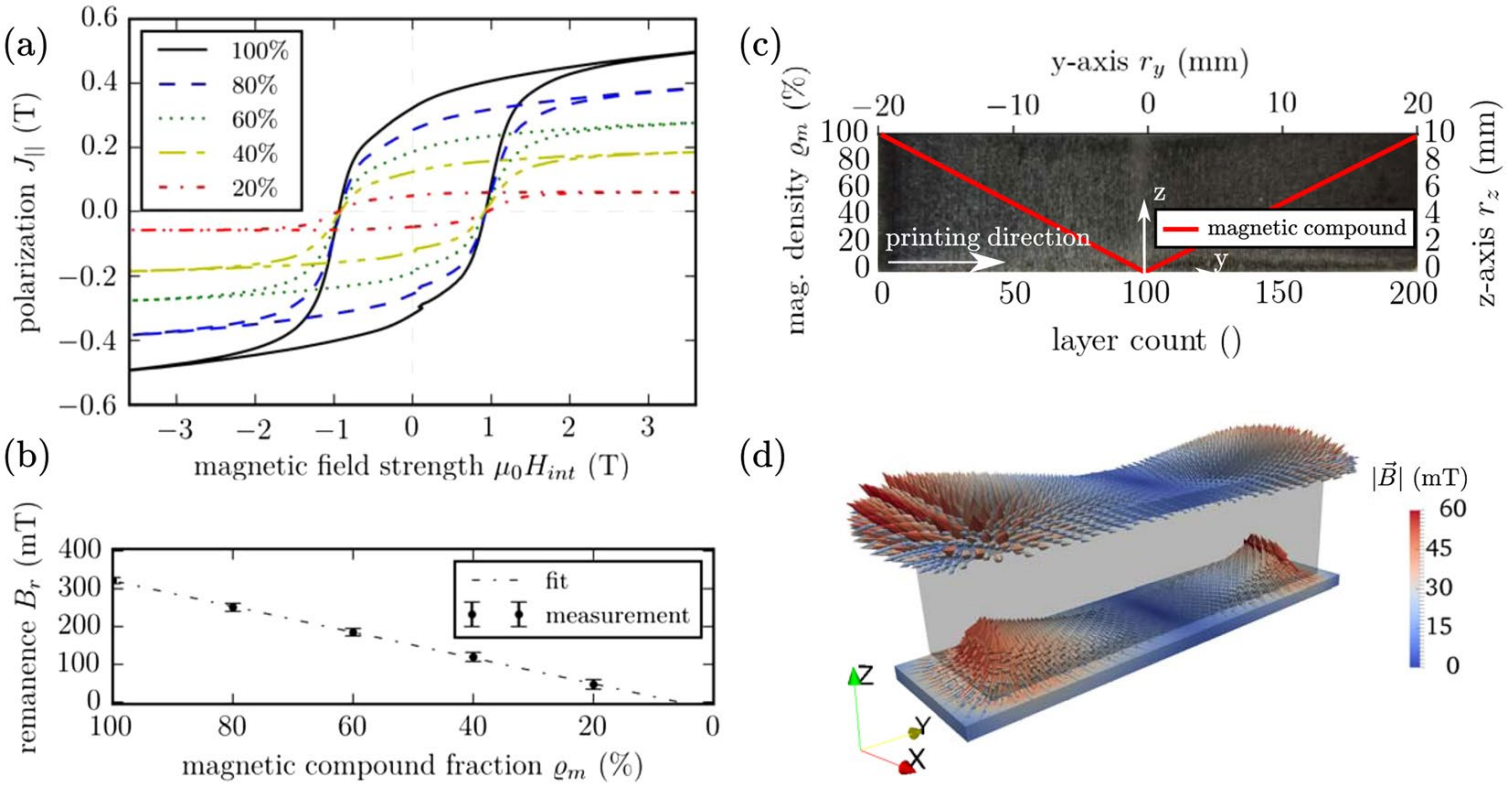
3D print of polymer-bonded magnets with a variable magnetic compound fraction. (**a**) Hysteresis measurements of the uniform distributed permanent magnetic powder (MQP-S-11-9) inside the PA12 matrix with different magnetic compound fractions ${\varrho }_{m}$. (**b**) Linear declining remanence ${B}_{r} \sim -{\varrho }_{m}$. (**c**) Picture of the printed cuboid (10 × 40 × 10 mm^3^ (L × W × H)), and the magnetization distribution along the *y*-axis *r*_*y*_. (**d**) Volume scan of the produced stray field above and under the printed magnet.

The mixing extruder can continuously change between both materials. The magnetic compound fraction is a function of the layer number and *y*-axis *r*_*y*_, respectively. To determine the magnetic properties of the prints with different magnetic compound filler fractions, hysteresis measurements of the uniform distributed NdFeB powder inside the PA12 matrix are performed and pictured in Fig. 1(a). Volumetric mass density measurements yield $\varrho =3.2$ g/cm^3^ for the maximum magnetic compound fraction of ${\varrho }_{m}=100 \%$. This is 15% lower than the theoretical volumetric mass density of the compound. With the FDM deposition technique, it is not possible to print complete dense objects. The circumstance that the high filler fraction of NdFeB particles reduces the flowability of the filaments, also decreases the maximum volumetric mass density of the printed objects. The compound exhibits a remanence *B*_*r*_ = 314 mT and a coercivity *H*_*cj*_ = 745 kA/m. However, the remanence *B*_*r*_ decreases linearly with the magnetic compound fraction ${\varrho }_{m}$ (Fig. 1(b)). This means that the maximum energy product ((*BH*_max_ ~ *B* ^2^)) is proportional to ${\varrho }_{m}^{2}$. To benchmark the variable magnetic compound fraction printing method, a cuboid of size 10 × 40 × 10 mm^3^ (L × W × H) with an absolute value magnetic distribution function $({\varrho }_{m}=100\, \% /(W\mathrm{/2)}|{r}_{y}|\, \% )$ is printed (Fig. 1(c)). The sample is magnetized inside an electromagnet with 1.9 T along the *z*-axis. A volume scan of the produced stray field above and under the magnetized cuboid is pictured in Fig. 1(d) ^8^. This measurement will be used to reconstruct the magnetization distribution inside the magnet and therefore deduct the quality of the printed magnet.

### Inverse Problem

The forward stray field computation problem is defined by finding the stray field for a given magnetization. Well-established finite element method (FEM) algorithms for the stray field calculation of permanent magnets exist^18^. In contrast to the forward problem, the inverse problem, where, for a given magnetic field outside the magnet, the magnetization within the magnet is reconstructed, is much harder to solve (Supplementary Fig. 5). The inherent difficulty of this inverse problem is due to the facts that (i) the inverse problem is not unique and (ii) the underlying system of equations is ill-conditioned. Mostly, no unique solution is available for these kinds of problems. A method to solve the inverse problem by using an adjoint method exists^19, 20^. In this article, a pure FEM method based on the FEM library FEniCS^13^, and the library dolfin-adjoint^21^ for the automatically derivation of the adjoint equation of a given forward problem is used. Dolfin-adjoint contains a framework to solve partial differential equation (PDE) constraint optimization problems.

The forward problem is a well-posed problem. This means a solution exists and that it is unique. As mentioned above, the inverse problem is ill-posed. To provide an approximated solution of the inverse problem, additional information is necessary. Different methods exists to find reasonable results^22^. Here, the Tikhonov regularization is implemented in the inverse stray field computation framework. Solving the following minimization problem results in the unknown magnetization $\overrightarrow{M}$ for each finite element of the model in the region Ω_*m*_ (Supplementary Fig. 1):

4 $$\mathop{{\rm{\min }}}\limits_{\overrightarrow{M}}({\int }_{{\Omega }_{h}}{\Vert {\overrightarrow{h}}_{{\rm{sim}}}-{\overrightarrow{h}}_{\exp }\Vert }^{2}\,{\rm{d}}\overrightarrow{r}+\mathop{\underbrace{\alpha {\int }_{{{\rm{\Omega }}}_{m}}{\Vert \nabla \overrightarrow{M}\Vert }^{2}\,{\rm{d}}\overrightarrow{r}}}\limits_{{\rm{regularization}}})$$

where ${\overrightarrow{h}}_{{\rm{sim}}}$ is the stray field calculated by the forward problem in a defined region Ω_*h*_, with the magnetic potential *u*. ${\overrightarrow{h}}_{\exp }$ is the measured or target stray field in the same region Ω_*h*_. *α* ≥ 0 is the Tikhonov regularization parameter. In this case, *α* has unit m^2^. The inverse problem is solved by means of a continuum approach. This being said, no individual particles are considered, but instead, the particle density is approximated by a continuous density field. As a consequence, there is no general restriction on the mesh size in order to retrieve reasonable results. We discretize all fields (${\overrightarrow{h}}_{{\rm{sim}}}$, ${\overrightarrow{h}}_{\exp }$, $\overrightarrow{M}$) with piecewise affine, globally continuous functions and apply the truncation method for the forward problem.

The main challenge for this regularization is the proper choice of a suitable parameter *α*. If *α* is too small, the solution will be dominated by the contributions from the data errors. If *α* is too large, the solution is a poor approximation of the original problem. A well-known method to find an optimal *α*, is the so-called L-curve method^23^. For this method, the solution norm ${\Vert \nabla \overrightarrow{M}\Vert }_{2}^{2}$ is plotted over the residual norm ${\Vert {\overrightarrow{h}}_{{\rm{sim}}}-{\overrightarrow{h}}_{\exp }\Vert }_{2}^{2}$ in a log-log scale for varying *α* ∈ [0, ∞). The optimal residual parameter *α* is where the curve has the maximum curvature *κ*_max_ (corner of the L-curve). This *α* value gives a good compromise between the change of the residual norm and the reduction of the solution norm (Supplementary Fig. 6). To solve the minimization problem in Eq. 4, the IPOPT software library for large-scale nonlinear optimization systems is used^24^.

### Reconstructed Magnetization

**Figure 2:**
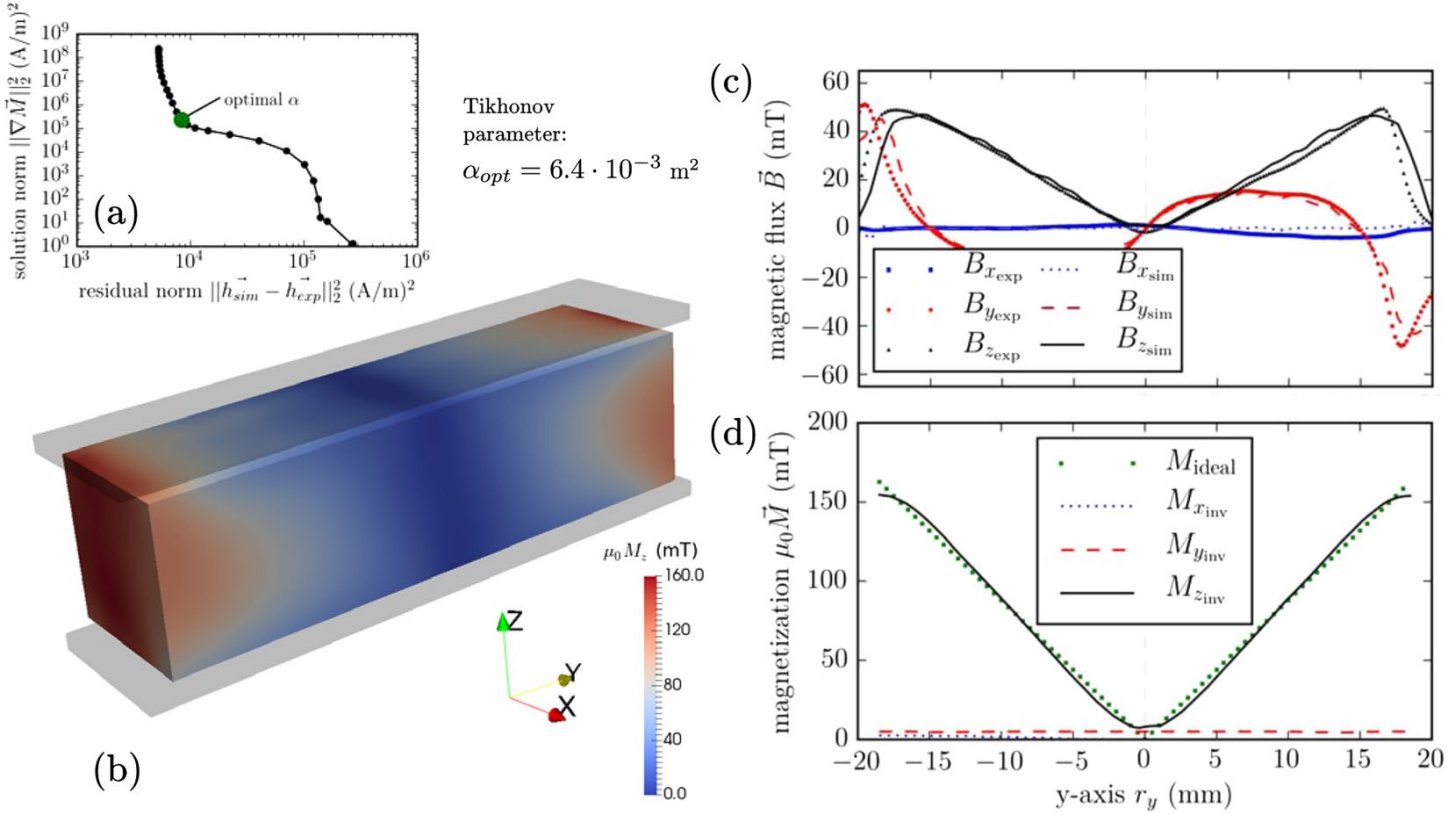
Reconstructed magnetization of a cuboid printed structure. (**a**) L-curve to find the optimal Tikhonov regularization parameter *α*. (**b**) Reconstructed magnetization distribution *μ*_0_ *M*_*z*_ of the magnet. (**c**) Line scan of the stray field 1.5 mm above the magnet compared with the inverse stray field simulation results. (**d**) Ideal magnetization in the middle of the magnet along the *y*-axis *r*_*y*_ compared with the reconstructed magnetization distribution.

To benchmark the inverse stray field framework and deduce the quality of the 3D printed magnetic cuboid with an absolute value magnetic distribution along the *y*-axis, the printed and magnetized magnet is scanned on both sides in a volume of 40 × 12 × 2 mm^3^ (L × W × H) with a spatial resolution of 0.2 mm in the magnetization direction *r*_*z*_ (Fig. 1(d)). The measured ${\overrightarrow{h}}_{\exp }$ is the input for the inverse stray field calculation. The simulation is performed for a range of different Tikhonov regularization parameters *α* = 10^*x*^ m^2^ with *x* ∈ [−9.4, 3] and a step size of 0.4. Regarding the error of the measurement, the L-curve looks different from the ideal one (Supplementary Fig. 6). First, the region with the optimal parameter *α* is limited; then, the maximum curvature *κ*_max_ in this region is calculated. Figure 2(a) shows the L-curve with the different *α* values, and the optimal solution with *α*_opt_ = 6.4 · 10^−3^ m^2^. The mesh of the simulation consists of 315,941 tetrahedral elements with a mesh size of the magnetic region of 0.25 mm. Figure 2(b) illustrates the magnetization distribution *M*_*z*_, which is proportional to the magnetic compound fraction distribution inside the magnet. The magnetization *M*_*z*_ is a function of *r*_*y*_ as expected, but it also depends on *r*_*z*_. In our case, only measurements under and above the magnet are used. Therefore, the algorithm cannot reconstruct the ideal distribution, and the print is not perfect. However, the example shows good conformity between the experimental and the simulation results. A line scan 1.5 mm above the magnet compared with the simulation results are shown in Fig. 2(c). It points out a good agreement between measurement and results from the inverse stray field calculation. The distribution along the *y*-axis in the middle of the magnet is plotted in Fig. 2(d). The reconstructed magnetization ${M}_{{z}_{{\rm{inv}}}}$ fits very well with the ideal magnetization distribution of ${M}_{{\rm{ideal}}}={M}_{{\rm{\max }}}/(W\mathrm{/2)|}{r}_{y}|$ mT or ${\varrho }_{m}=100\, \% /(W\mathrm{/2)|}{r}_{y}|$ % for the magnetic compound fraction distribution, where *M*_max_ is the maximum magnetization and *W* is the width of the magnet. The reconstructed components ${M}_{{x}_{{\rm{inv}}}}$ and ${M}_{{y}_{{\rm{inv}}}}$ are small compared to the z component. This complies with the expectations of the printed permanent magnet. A supplementary animation shows the change of the magnetization distribution and the resulting stray field at different Tikhonov regularization parameters *α*. If *α* → 0, the magnetic compound fraction ${\varrho }_{m}$ distribution is unphysical, but the stray field fits with the measurement data. If *α* → ∞, ${M}_{{z}_{{\rm{inv}}}}=1$ mT for the whole magnetic region, and therefore, the stray field above the magnet is mismatched with the measurement data.

### Predefined Stray Field

**Figure 3:**
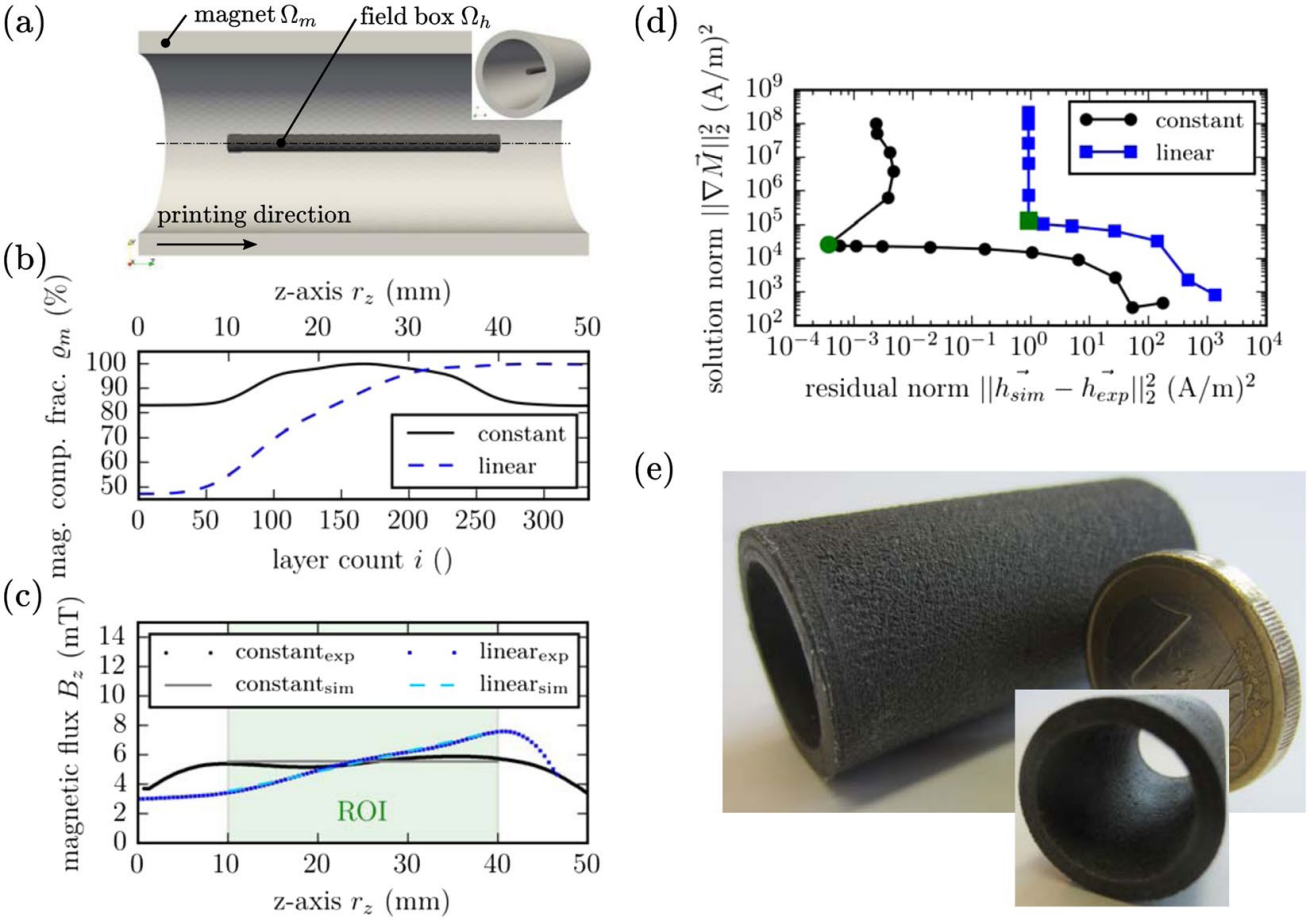
3D prints of magnetic hollow cylinder with a variable magnetic compound fraction distribution to generate a predefined stray field inside the cylinder. (a) Model of the hollow cylinder magnet with the dimension in mm (∅25, ∅20, 50 (*d*_outer_, *d*_inner_, *L*)) with a predefined stray field in the field box (∅2, 30 (*d*, *L*)). (**b**) Magnetic compound fraction distribution ${\varrho }_{m}$ along the *z*-axis *r*_*z*_ to create a constant and linear stray field in the field box, respectively. (**c**) Stray field measurements of *B*_*z*_ compared with inverse stray field FEM simulations in the middle of the hollow cylinder for the linear and constant field generations magnet, respectively. (**d**) L-curve for both designs to find the optimal Tikhonov regularization parameter *α*. (**e**) Picture of the hollow cylindrical magnet.

Instead of using the inverse stray field method to investigate already printed magnets, the method can also be used to design magnets with specific stray field properties. As examples, we compute the optimal magnetization distribution for a hollow cylinder geometry for different target fields inside the cylinder. The hollow cylinders have the dimension in mm ∅25, ∅20, and 50 (*d*_outer_, *d*_inner_, *L*) with a linear and constant stray field distribution inside the hollow magnet. Figure 3(a) shows the model of the magnet with the magnetic region Ω_*m*_ and the region for the predefined stray field Ω_*h*_. The mesh Ω of the simulation consists of 455,306 tetrahedral elements with a mesh size of the magnetic region Ω_*m*_ of 0.4 mm. The printing direction is along the *z*-axis. For this reason, the variable ${\overrightarrow{h}}_{\exp }$ in Eq. 4 does not represent the measurement data but rather the desired stray field in distributions Ω_*h*_. *M*_*x*_ and *M*_*y*_ are fixed to zero, and the maximum of *M*_*z*_ is limited to the used magnetic material. Otherwise, the real printed magnet cannot reach the desired magnetization. Two different stray field distributions are tested. The first is a constant magnetic flux density of *B*_*z*_ = 5.5 mT along the *z*-axis *r*_*z*_ ∈ [10, 40] mm; the second one is a linear increasing field of *B*_*z*_ = 2 + 0.15*r*_*z*_ mT/mm along the *z*-axis *r*_*z*_ ∈ [10, 40] mm. A constant magnetic field inside a hollow cylinder can be used to calibrate sensors where the sensor position is changing. A linear increasing field can be used to realize a linear positioning system. In this case, a 1D sensor is enough for an accurate position detecting system^25^. The resulting magnetic compound fraction distribution along the *z*-axis is plotted in Fig. 3(b). Figure 3(c) shows the comparison between simulations and measurements in the middle of the hollow cylinders. The inverse stray field simulation for both examples is performed for various Tikhonov regularization parameter *α* = 10^*x*^ m^2^ with *x* ∈ [−10, 1]. The L-curve for both simulations is presented in Fig. 3(d). *α*_opt_ is clearly visible and is marked in green (*α*_opt_ = 2.5 · 10^−7^ m^2^ for both designs). Inside the field boxes with the dimensions in mm of ∅2, 30 (*d*, *L*), a good conformity between printed and simulated magnets is given. A picture of one of the printed magnets is presented in Fig. 3(e).

**Figure 4:**
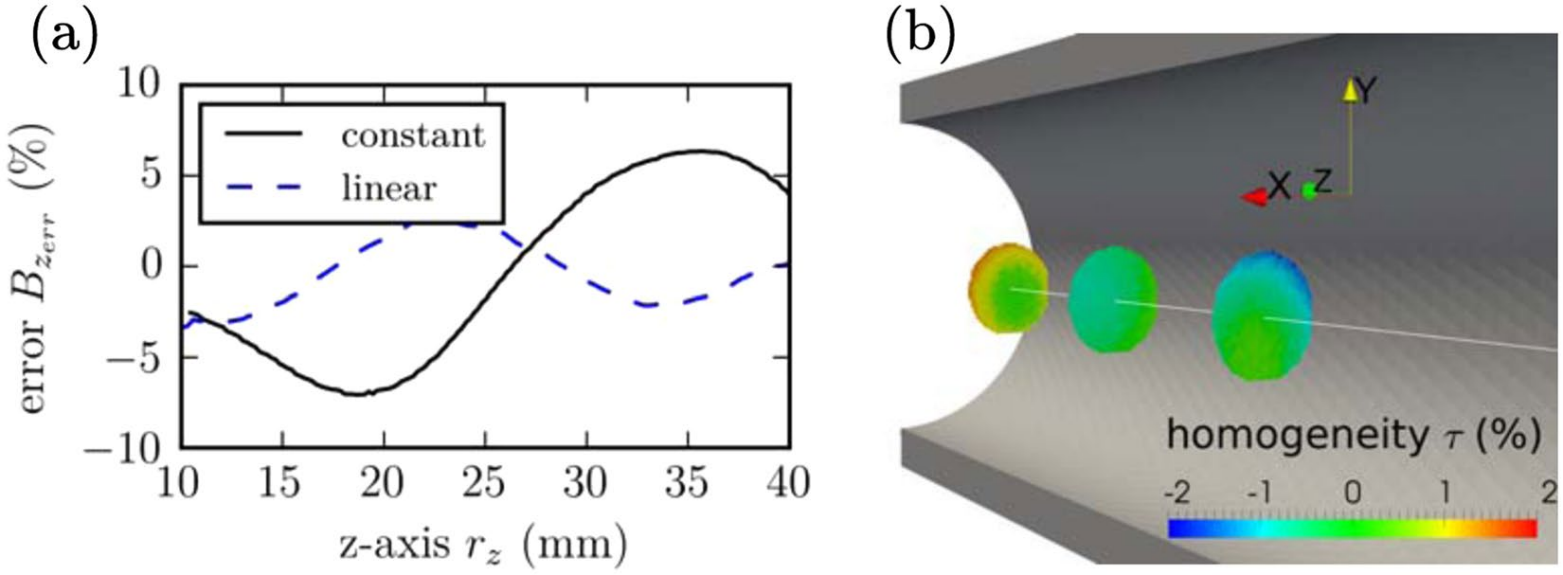
Errors of the printed magnets for a predefined stray field. (**a**) Error between the measured stray field and the inverse stray field simulation along the *z*-axis *r*_*z*_ for the linear and constant stray field generator magnets. (**b**) Homogeneity *τ* within a radius of *r* = 2.5 mm on three planes (*r*_*z*_ = 15, 25, 35 mm).

The error between measurements and simulations is plotted in Fig. 4(a). The error changes along the *z*-axis, and it is around 6% for the constant and 4% for the linear design. Another important feature of this magnetic design is the homogeneity of *B*_*z*_. The homogeneity is defined as $\tau =({B}_{z}(r)/{B}_{z}\mathrm{(0)}-\mathrm{1)100 \% }$. Figure 4(b) shows a plot of the homogeneity *τ* within a radius of *r*_*z*_ = 2.5 mm on three planes (*r*_*z*_ = 15, 25, 35 mm) inside the hollow cylinder. The deviation of the homogeneity is lower than 2%.

## Summary

Additive manufacturing of polymer-bonded magnets has the advantage of manufacturing magnets with a minimum of cost and time. This article presents a method to 3D print permanent magnets with a variable magnetic compound fraction distribution along the printing direction. With a commercially available end-user 3D printer and a mixing extruder, a polymer-bonded magnetic filament can be mixed with a pure PA12 filament. Hysteresis measurements with different magnetic compound filler fractions are performed to get a relation of the remanence and magnetic compound fraction.

To deduct the quality of the prints, an inverse stray field simulation framework is developed. No unique solution exists for this kind of inverse problem. Therefore, Tikhonov regularization is used to find reasonable results for the optimization problem. A cuboid with an absolute value function of the magnetic density is printed, and the inverse stray field code is benchmarked with some measurements.

The inverse stray field computation framework can be used to simulate magnets with a predefined target stray field in a given region outside of the magnet. The optimal magnetization density of a hollow cylinder for a targeted constant or linear stray field is computed. The magnetic compound fraction distribution along the *z*-axis is optimized and printed with our setup. Detailed stray field measurements show excellent agreement between the simulation and measurement. Homogeneity of the field is an important aspect for linear position measurement systems. Our examples show a low dependence of an eccentric sensor position.

With this printing setup and simulation framework, the manufacturing of magnets is possible, which are impossible to create with conventional methods. It can be used to create magnets with a specific stray field distribution for various applications. The simulation method can also be used to improve the performance of multipolar polymer-bonded magnets by injection molding.

At the moment, only prints with a variable magnetic compound fraction along the *z*-axis are possible. An improved slicing program should rescind this restriction. An influence on the quality of the printed structures is the filament diameter, because with a constant feeding rate, the volume flow through the nozzle varies, which leads to a patchy printing result. The deviation of the remanence due to the changing filament diameter is around 3%.

The deviation of the ideal volumetric mass density and the printed one of around 15% is a result of the flowability of the material and the FDM technique itself. Here is a potential for improvement of the printing results, and to reduce the error between simulations and measurements.

## Electronic supplementary material

Supplementary Info

Animation of the alpha-sweep
