# Supplementary material for: 3D Printing of Polymer-Bonded Rare-Earth Magnets With a Variable Magnetic Compound Fraction for a Predefined Stray Field
Source: Sci Rep. 2017 Aug 25;7:9419. doi: 10.1038/s41598-017-09864-0 (PMC5572745; doi:10.1038/s41598-017-09864-0)
Supplement: Supplementary file 1 — Supplementary Info [file 41598_2017_9864_MOESM1_ESM.pdf]

# Supplementary Informations for "3D Printing of Polymer-Bonded Rare-Earth Magnets With a Variable Magnetic Compound Fraction for a Predefined Stray Field"

**Christian Huber[1,2,*], Claas Abert[1,2], Florian Bruckner[1,2], Martin Groenefeld[3], Stephan Schuschnigg[4], Iulian Teliban[3], Christoph Vogler[1], Gregor Wautischer[1,2], Roman Windl[1,2], and Dieter Suess[1,2]**

[1]Physics of Functional Materials, University of Vienna, 1090 Vienna, Austria
[2]Christian Doppler Laboratory for Advanced Magnetic Sensing and Materials, 1090 Vienna, Austria
[3]Magnetfabrik Bonn GmbH, 53119 Bonn, Germany
[4]Department of Polymer Engineering and Science, Montanuniversitaet Leoben, 8700 Leoben, Austria
[*]huber-c@univie.ac.at

## Supplementary Movie

The animation shows the dependence of the magnetization distribution $\vec{M}$ from the Tikhonov regularization parameter $\alpha$. It starts with a low $\alpha$ parameter where the solution is dominated by the contributions from data errors. At this stage, the stray field fits well to the measured field, but the magnetization distribution is nonphysical. At the optimal regularization parameter $\alpha_{\text{opt}}$ the magnetization fits to the predicted distribution, and the stray field above the magnet fits to the measurement data. If $\alpha$ is too large, the solution is a poor approximation of the original problem. The reconstructed magnetization approximates a uniform distribution and therefore, the stray field above the magnet mismatch with the measurement data.

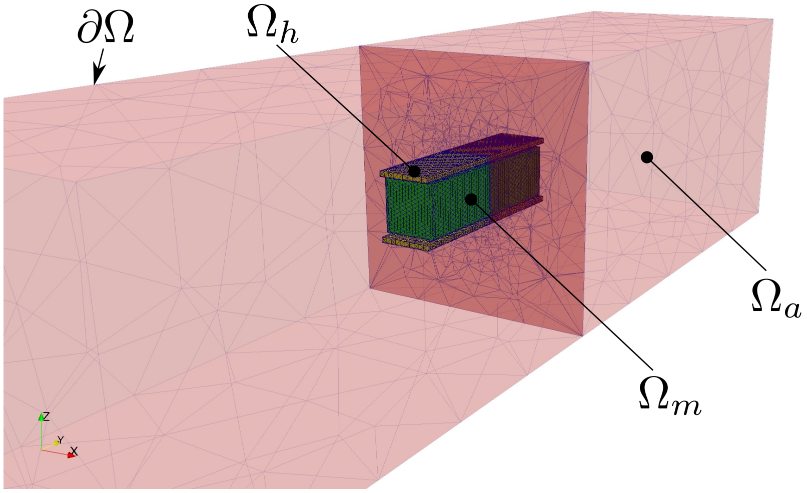


**Supplementary Figure 1.** Different regions of a magnetic system. $\Omega_m$ is the magnetic region with a magnetization $\vec{M}$. $\Omega_m$ describes a stray field box. For the inverse stray field calculation the error between the forward stray field calculation and the predefined or measured field $\vec{B}$ in this field box is minimized. To fulfill the Neumann boundary condition $u = 0$ at $\partial\Omega$, the air box $\Omega_a$ should be five-times larger as the magnetic region to get accurate solutions. To minimize the computing time the mesh size increase from the magnetic region to the end of the air region with $u = 0$ at $\partial\Omega$.

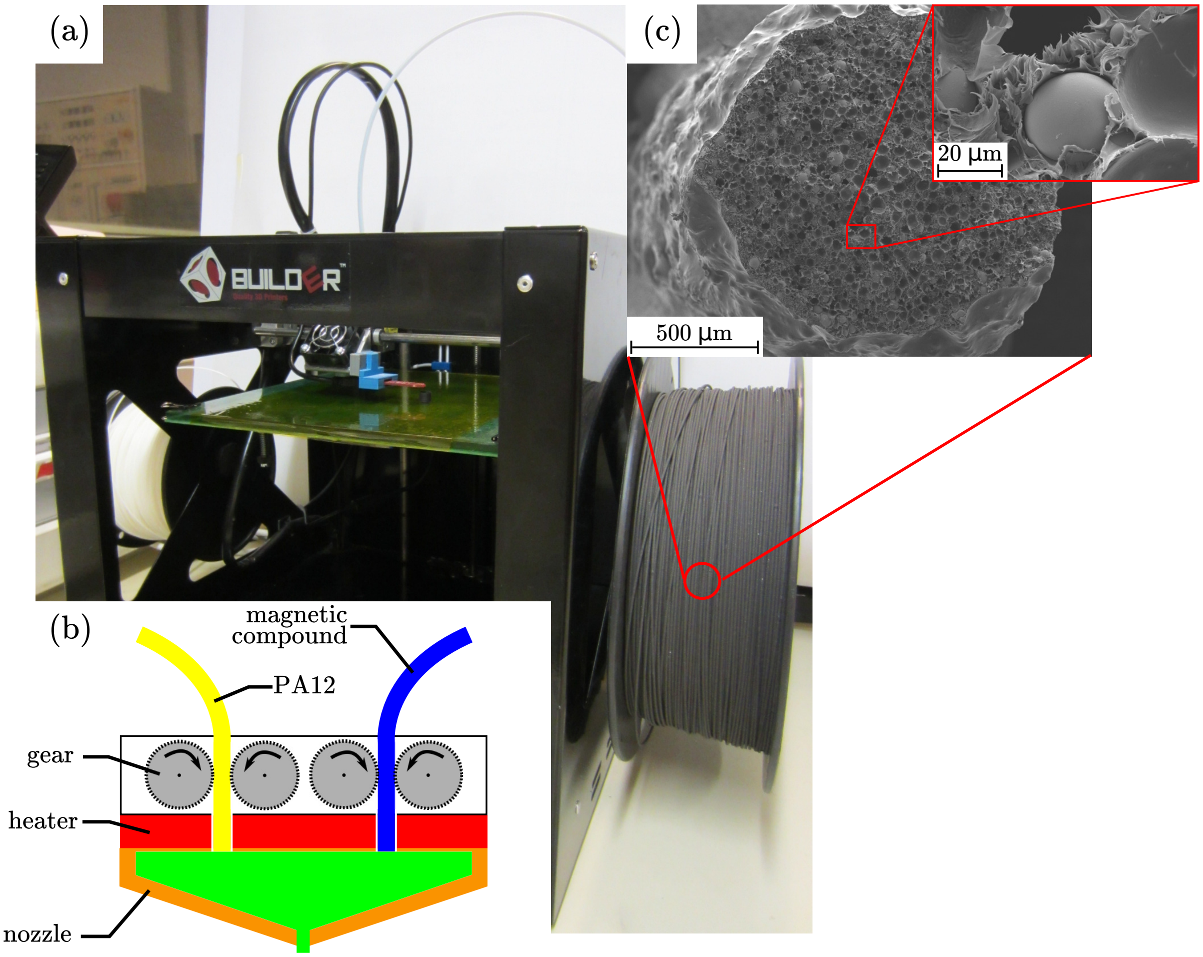


**Supplementary Figure 2.** 3D printer setup. (a) Builder 3D printer from Code P. (b) Sketch of the dual feed extruder to mix two different materials. (c) Coiled magnetic filament.

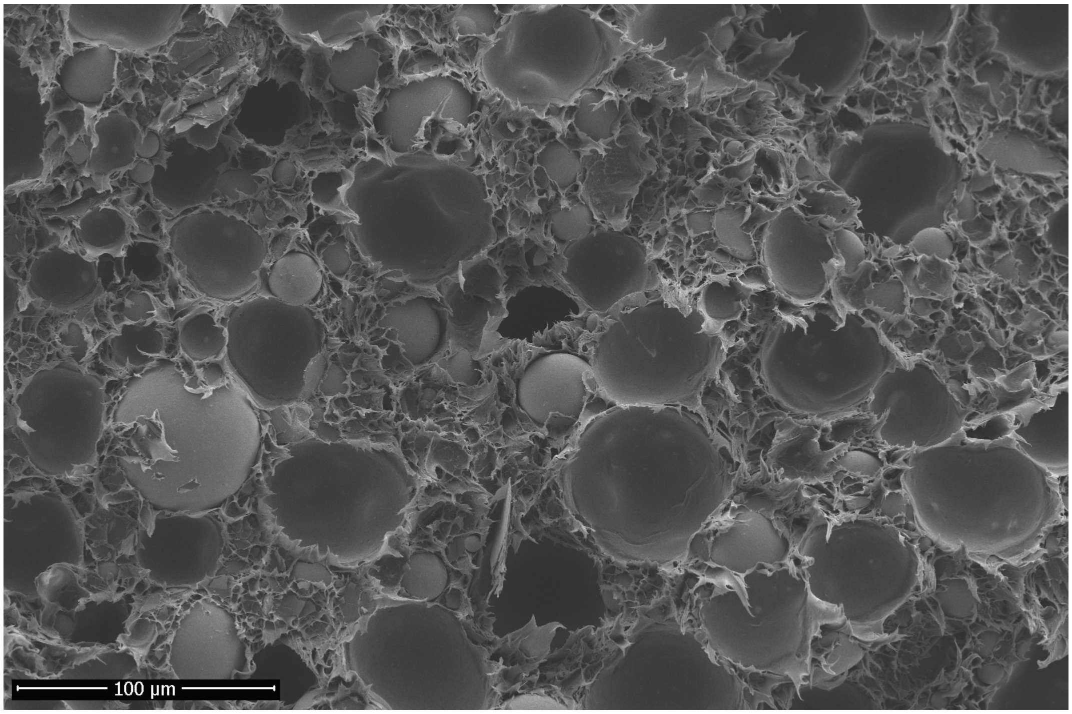


**Supplementary Figure 3.** Scanning electron microscope image of the manufactured filament to identify the morphology and the size of the NdFeB particles (MQP-S-11-9) inside the PA12 matrix.

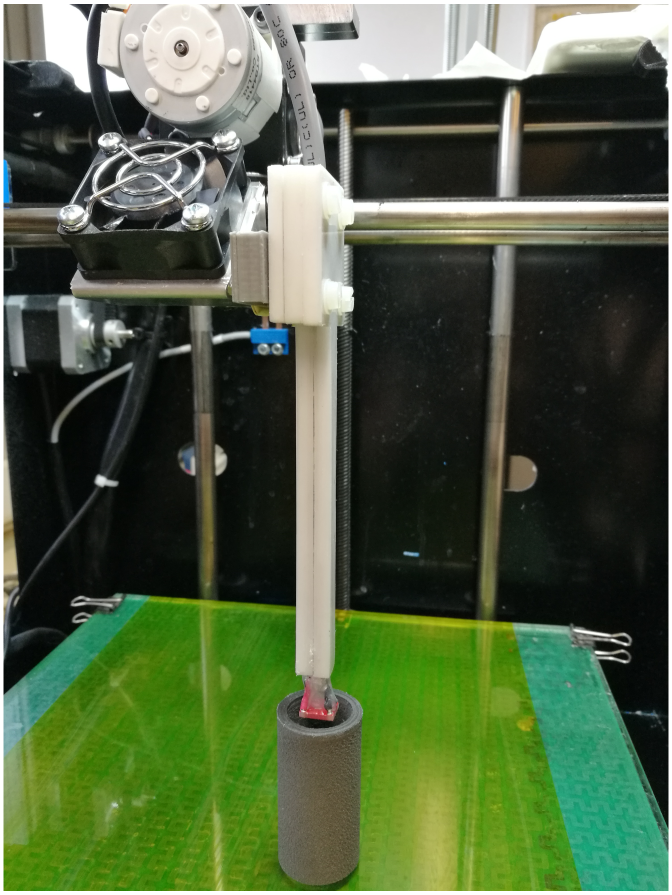

**Supplementary Figure 4.** Setup to measure the stray field around magnets. Self printed suspension is directly attached to the printer head.

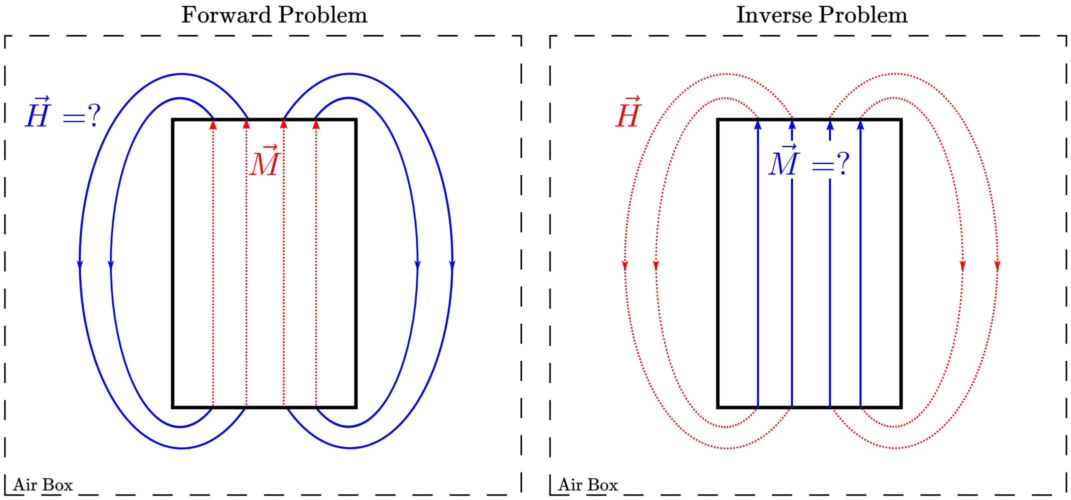


**Supplementary Figure 5.** The forward problem computes the stray field $\vec{H}$ of a given object with a magnetization $\vec{M}$. This is a well-posed problem, it exists an unique solution of this problem. Easy to compute with modern FEM simulation algorithms. Compared to the forward problem is the inverse stray field problem. Here, outgoing from a predefined stray field $\vec{H}$ (i.e. a 3D measurement in a defined region outside the magnet) the magnetization $\vec{M}$ should be calculated. It is an ill-posed problem and therefore, no exact solution exists. To solve this problem a regularization method is necessary.

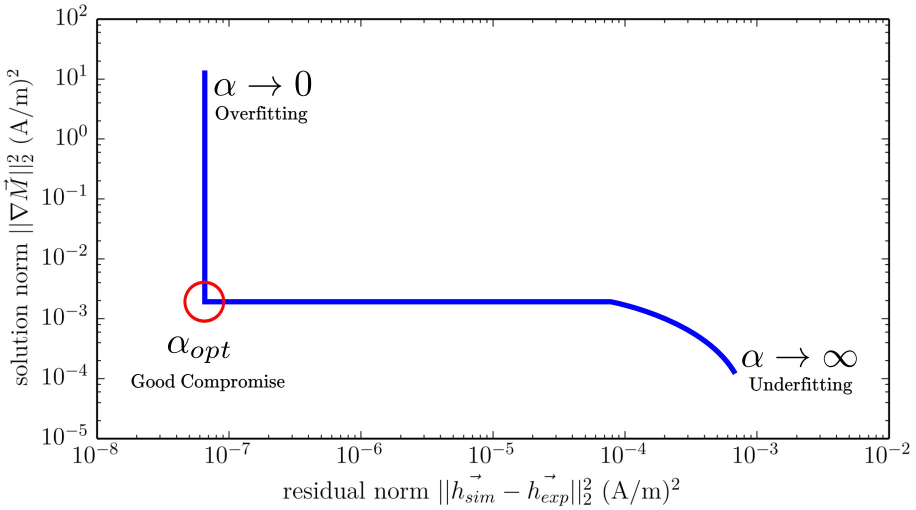


**Supplementary Figure 6.** Theoretical L-curve to determine the best choice for the regularization parameter $\alpha$. At the corner the curvature of the L-curve is maximal which identify best compromise between noise in the residual norm and a poor approximation of the original problem.

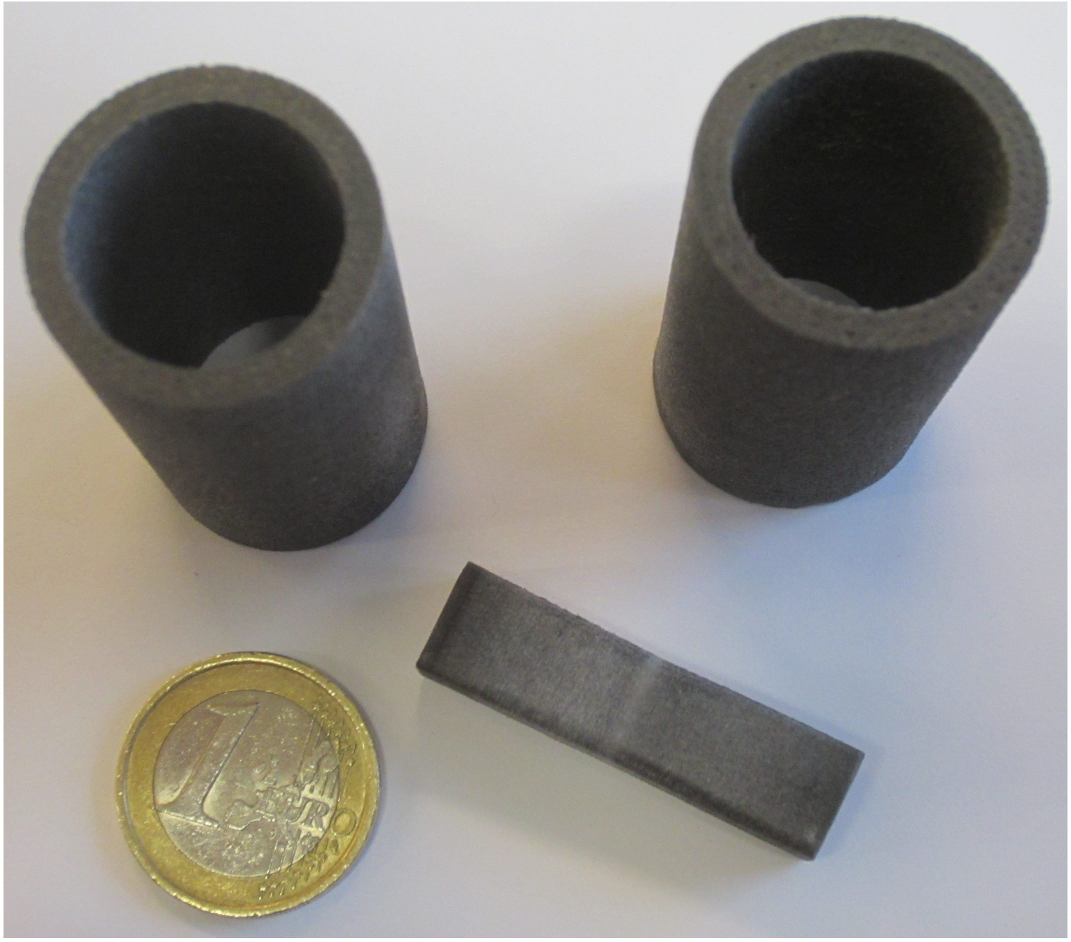

**Supplementary Figure 7.** Printed magnets for this article. (front) Magnet with an absolute value distribution of the magnetic compound denity. (left) Magnetic hollow cylinder with a linear increasing stray field inside the cylinder. (right) Magnetic hollow cylinder with a constant stray field inside the cylinder.
